# Methodology and predictive accuracy of the prospective preference assessment for randomized trial enrollment: A literature review

**DOI:** 10.1016/j.ocarto.2025.100696

**Published:** 2025-10-15

**Authors:** Jon S. Dhani, Faith Selzer, Jamie E. Collins, Katharine B. Fox, Paul Oh, Elena Losina, Jeffrey N. Katz

**Affiliations:** a The Orthopaedic and Arthritis Center for Outcomes Research, Brigham and Women's Hospital, 75 Francis St, Boston, MA 02115, USA; b Harvard Medical School, 25 Shattuck Street, Boston, MA 02115, USA; c Harvard School of Public Health, 677 Huntington Ave, Boston, MA 02115, USA

**Keywords:** Prospective preference assessment, PPA, RCT, Feasibility, Willingness

## Abstract

**Objective:**

The success of a randomized controlled trial (RCT) depends, in part, on the willingness to participate (WTP) of eligible subjects. Prospective preference assessments (PPA) offer insights into future RCT enrollment by evaluating eligible individuals’ WTP in the RCT. We sought to summarize the methods and findings of published PPAs and to compare the WTP estimates of published PPAs to the actual enrollment rates from these trials.

**Design:**

We performed a systematic literature search using PubMed for studies that assessed eligible participants’ WTP in a hypothetical trial. We abstracted sample size, proposed interventions, WTP response options, WTP percentage, presence of qualitative analyses, and assessment of trial feasibility from each publication. We defined WTP as a response of “probably” or “definitely” willing in the PPA; in sensitivity analyses, we included only “definitely” willing responses. We searched for registered and published RCTs connected to each PPA and extracted enrollment data from the corresponding trial publications where available.

**Results:**

We identified 40 PPAs. The median WTP across all PPAs was 54.9 ​% (range: 13 ​%–92.4 ​%), and 42.1 ​% (range: 7 ​%–90.2 ​%) when including only “definitely willing” responses. We found ten registered RCTs; five are completed and one is ongoing. Four PPAs with a RCT provided both “definitely willing” and “total willing” estimates. In three of these four RCTs, the actual enrollment fell between the “definitely willing” and “total willing” PPA estimates.

**Conclusion:**

“Definitely willing” and “total willing” findings in a PPA may provide useful upper and lower boundaries on participation for future trial planning.

## 1 Introduction

Randomized Controlled Trials (RCTs) are among the most scientifically rigorous study designs for assessing treatment efficacy, but enrollment into RCTs often poses challenges [1,2]. A lack of willingness to participate (WTP) in an randomized controlled trial (RCT) can significantly lengthen the duration of the enrollment period and introduce a selective enrollment bias, leading to outcomes that are no longer generalizable to the eligible population. Prior to trial initiation, investigators may conduct a prospective preference assessment (PPA) to assess the willingness of eligible subjects to participate in the trial [3]. In a PPA, potential participants are presented with a hypothetical trial and asked whether they would participate if the trial were offered. The results of a PPA can be used to question the feasibility of conducting an RCT, estimate subject enrollment, and identify features of subjects more or less likely to participate.

We aimed to provide a narrative review of studies that assessed participants’ WTP in hypothetical RCTs. We highlight the methodological details of these studies. To gain insight into whether PPAs were followed by implementation of the trial that the PPA characterized, we identified whether each PPA was associated with a trial registered in a clinical trials database. Furthermore, among the PPAs with a completed or published RCT, we compared the estimates of subject WTP documented in the PPA with actual trial enrollment data to gain insight into the predictive accuracy of PPAs.

## 2 Methods

### 2.1 Search process

We identified studies in which investigators planning an RCT queried potential participants’ WTP in a hypothetical version of the planned trial. We conducted a PubMed search on October 25th, 2023, for studies published between 1966 and 2023, using two sets of search terms. The first search used the terms: “WTP” AND (“survey” OR “questionnaire”). We used papers from the first query to identify keywords that would elicit additional manuscripts in a second search. Our second search query used the terms: “hypothetical” AND “participation” AND (“willingness” OR “agree”) AND (“randomized trial” or “randomised trial”) OR “prospective preference assessment.” We aggregated search results from both queries and removed duplicates.

### 2.2 Exclusion criteria and process

We excluded from further consideration papers that met one or more of our exclusion criteria. These included: WTP in a hypothetical RCT was not assessed quantitatively; preferences were not assessed prospectively; and data were not gathered from subjects who were potential participants in the planned RCT. We performed exclusions based on the title and abstract and obtained full manuscripts of papers not excluded at this phase. We then read the full manuscript and excluded papers that met any of the exclusion criteria, as detailed above. We included one unpublished study from our own group (manuscript in preparation) that met eligibility criteria. Two reviewers assessed the titles, abstracts and papers separately and arrived at the same set of eligible manuscripts.

### 2.3 Data elements

We abstracted the following information from eligible papers: author and year, journal, sample size, the proposed intervention of the hypothetical RCT, willingness response options, and the percentage of individuals indicating they would participate in the proposed clinical trial. We reported WTP with the response categories used in each study. For example, many studies asked subjects if they were “definitely willing,” “probably willing,” “probably not willing,” etc. We aggregated “definitely willing” and “probably willing” as “total willing” in our analysis. We performed sensitivity analyses restricting the definition of willingness to “definitely willing” for trials that provided this response option. Trials with a singular willing option (e.g. yes/no, agree/unsure/disagree) were also included in the sensitivity analysis. We calculated the median and range of willingness across all studies and within two distinct intervention pairings: operative vs. non-operative and drug vs. placebo. We also documented if a paper included a qualitative portion within their PPA, and if the authors of the PPA commented on the feasibility of conducting a trial based on PPA data.

To determine the predictive accuracy of the PPA methodology, for each PPA in our sample, we searched for a clinical trial that was registered and/or completed. We used ClinicalTrials.gov and PubMed to search for a registered clinical trial number or publication related to the PPA's author(s), site location, research group, and proposed intervention. We also used ClinicalTrials.gov and PubMed to identify PPA-connected trial publications showing trial results, protocol design, or abstracts. We included a trial from our own research group that published a PPA and completed enrollment but is not yet published. We calculated the proportion of eligible participants who were randomized in these trials by abstracting the enrollment data (often presented in a CONSORT diagram) in the trial publications. We calculated 95 ​% confidence intervals around the estimates of WTP in the PPAs and around the actual randomization rates reported in the trials using a binomial distribution based on reported sample size data. We compared WTP data between PPAs connected to a registered RCT and PPAs not connected to a registered RCT.

## 3 Results

### 3.1 Search results

**Fig. 1:**
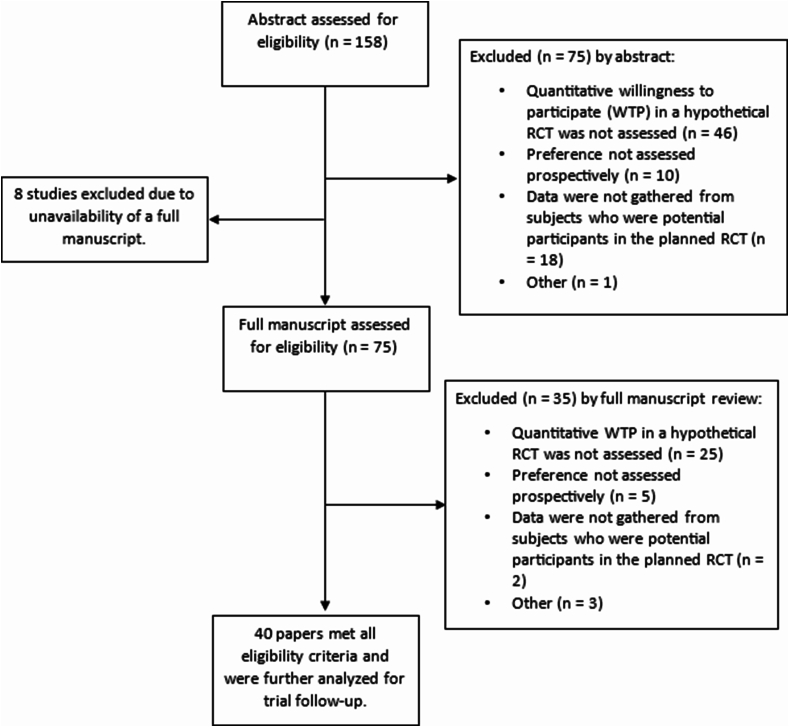
Flowchart of search process and exclusion criteria from the two searches: “WTP” AND (“survey” OR “questionnaire”), “hypothetical” AND “participation” AND (“willingness” OR “agree”) AND (“randomized trial” OR “randomised trial”) OR “prospective preference assessment.”

The search queries yielded 158 unique publications. We (JD, PO) reviewed the titles and abstracts of these 158 publications and eliminated 75 meeting one or more exclusion criteria (Fig. 1). The most frequent reasons for papers to be excluded at the title and abstract stage were that the paper did not assess WTP quantitatively in a hypothetical RCT (n ​= ​46, 29 ​%), the data were not gathered from subjects who were potential participants in the planned RCT (n ​= ​18, 11 ​%), and preference was not assessed prospectively (n ​= ​10, 6 ​%). Eight papers were eligible for review at the full manuscript stage, but the full manuscript was not accessible electronically or with our university reference library services. We reviewed the full manuscripts of 75 papers and using the same exclusion criteria, we narrowed the eligible pool down to 40 papers. The most frequent exclusion at the full manuscript stage was the paper did not assess WTP quantitatively in a hypothetical RCT (n ​= ​25, 33 ​%).

### 3.2 Features of PPAs

Table 1 depicts the 40 PPAs and highlights features including journal, sample size, intervention proposed, the response options for the WTP question, and the percentage of subjects willing to participate. The median sample size was 155.5, with a range between 20 and 1193 subjects. Ten papers asked participants about their WTP in an RCT without any specified intervention. For example, Jenkins et al. asked its participants, “Would you be prepared to take part in a study where treatment was chosen at random?” We categorized these studies as “No Specified Intervention.” For willingness response options, the most common scale types were binary (e.g. willing vs. unwilling; n ​= ​14, 35 ​%), 5-point ordinal Likert scale (e.g. definitely willing, probably willing, unsure, probably not, definitely not; n ​= ​12, 30 ​%), and 3-item scale (e.g. yes, no, don't know; n ​= ​9, 23 ​%). Seven papers [[4], [5], [6], [7], [8], [9], [10]] included a qualitative portion to their PPA either through interviews or open-ended responses to the questionnaire. Three of the seven qualitative appeared to aid trial development. Specifically, in Walker et al. the qualitative results suggested to investigators that a trial was feasible, and two PPAs with qualitative analyses went on to registered trials [6,7].

**Table 1:** Features of eligible PPAs.

First Author, Year	Journal	Sample Size (n)	Interventions proposed	Willingness Response Options	Willing to Participate [95 ​% CI]
Abboud PA, 2006 [23]	J Med Ethics	345	Standard vs. new drug without informed consent	Agree/Disagree	60.3 ​% agree [55.1, 65.5]
Agoritsas T, 2011 [51]	J Clin Epidemiol	1172	No Specified Intervention	5-point Likert scale (certainly agree, probably agree, …, certainly disagree)	N/A^a^
Boye GN, 2015 [11]	Orthop J Sports Med	75	Non-operative vs operative treatment of femoroacetabular impingement	5-point Likert scale (definitely, probably, …, definitely not)	28.0 ​% definitely [17.8, 38.2]
					30.7 ​% probably [20.3, 41.1]
Cescon DW, 2012 [19]	Breast Cancer Res Treat	173	Vitamin D vs placebo for breast cancer	Yes/No	68.2 ​% yes [61.3, 75.1]
Cho MK, 2015 [52]	Ann Intern Med	1095	No Specified Intervention^b^	Yes/No	72.9 ​% yes [70.3, 75.5]
Christopher PP, 2017 [47]	PLoS One	45	No Specified Intervention	Yes/No	56.3 ​% yes [41.8, 70.8]
Creel AH, 2005 [12]	Contemp Clin Trials	88	Non-operative vs operative treatment of meniscal tears	5-point Likert scale (definitely willing, probably willing, …, definitely not)	22 ​% definitely willing [13.3, 30.7]
					24 ​% probably willing [15.1, 32.9]
Croll DMR, 2022 [24]	Eur J Obstet Gynecol Reprod Biol	70	Induction of labor vs Expected management for nulliparous women	3-item scale (would participate, might participate, would not participate)	15.7 ​% would participate [7.2, 24.2]
					24.3 ​% might participate [14.3, 34.3]
De Biase G, 2022 [25]	World Neurosurg	50	Spinal vs general anesthesia for spinal surgery	5-point Likert scale (definitely willing, probably willing, …, definitely not)	52 ​% definitely willing [38.2, 65.8]
					8 ​% probably willing [0.5, 15.5]
Ding EL, 2007 [20]	Arch Intern Med	400 women 383 men	Placebo vs drug in cardiovascular disease prevention trials	5-point Likert scale (very likely, likely, …, very unlikely)	Reported very likely or likely:
					28.7 ​% women [24.3, 33.1]
					33.1 ​% men [28.4, 37.8]
Dolan L, 2008 [26]	J Bone Joint Surg Am	24	Bracing vs observation for adolescent idiopathic scoliosis	Agree/Refuse	13 ​% agree [0, 26.5]
Ellis PM, 2002 [27]	Ann Oncol	30	Tamoxifen vs chemotherapy for breast cancer	3-item scale (would, unsure, would not)	46.7 ​% would [28.8, 64.6]
Gaudiano BA, 2016 [53]	Prim Care Companion CNS Disord	596	Antidepressants vs psychotherapy for depression	10-point Likert scale (1 ​= ​definitely not willing to participate, …, 10 ​= ​definitely willing to participate)	N/A^c^
George DE, 2018 [4]	Indian J Med Ethics	20	No Specified Intervention	3-item scale (agree, unsure, disagree)	55 ​% agree [33.2, 76.8]
Gharaibeh L, 2020 [54]	Int J Clin Pharm	1193	No Specified Intervention	3-item scale (yes, do not know, no)	64.4 ​% yes [61.7, 67.1]
Halpern SD, 2003 [5]	Am Heart J	126	Placebo vs. new hypertensive drug	6-point Likert scale (definitely would, probably would, maybe would, …, definitely would not)	9 ​% definitely would [4.0, 14.0]
					15 ​% probably would [8.8, 21.2]
Hyung B, 2022 [6]	Trials	42	Opioid-based vs opioid-sparing therapy for postoperative pain	6-point Likert scale (definitely, probably, maybe, …, definitely not)	17 ​% definitely [5.6, 28.4]
					43 ​% probably [28.0, 58.0]
Igwe E, 2016 [35]	Gynecol Oncol	101	No Specified Intervention	3-item scale (yes, do not know, no)	61.4 ​% yes [51.9, 70.9]
Israni AK, 2004 [28]	Kidney Int	209	Daily vs conventional hemodialysis	6-point Likert scale (definitely would, probably would, maybe would, …, definitely not willing)	7 ​% definitely [3.5, 10.5] 17 ​% probably [11.9, 22.1]
Jaffe K, 2021 [45]	Subst Abus	229	No Specified Intervention	5-point Likert scale (definitely, probably, …, definitely not)	62.0 ​% definitely [55.7, 68.3]
					22.7 ​% probably [17.3, 28.1]
Jenkins V, 2010 [46]	Br J Cancer	1066	No Specified Intervention	3-item scale (yes, do not know, no)	55.3 ​% yes [52.3, 58.3]
Kasiske BL, 2011 [7]	Am J Kidney Dis	212	Routine screening vs no screening for coronary artery disease	5-point Likert scale (definitely yes, probably yes, …, definitely not)	47 ​% definitely yes [40.3, 53.7]
					28 ​% probably yes [22.0, 34.0]
Kerman HM, 2017 [29]	Contemp Clin Trials Commun	134	Home exercise vs in-person physical therapy vs topical treatment for meniscus tear treatment	5-point Likert scale (definitely willing, probably willing, …, definitely not)	24 ​% definitely willing [16.8, 31.2]
					39 ​% probably willing [30.7, 47.3]
Kerr CE, 2004 [8]	Patient Educ Couns	113	No Specified Intervention	3-item scale (yes, do not know, no)	77.9 ​% yes [70.2, 85.6]
Kim SY, 2013 [30]	PLoS One	503	1) Vaccine RCT for Alzheimer's 2) New drug treatment RCT for Alzheimer's	4-item scale (definitely yes, probably yes, …, definitely no)	Vaccine RCT:
					16.7 ​% definitely yes [13.4, 20.0]
					38.0 ​% probably yes [33.8, 42.2]

					Drug RCT:
					48.9 ​% definitely yes [44.5, 53.3]
					43.5 ​% probably yes [39.2, 47.8]
Maisonneuve AS, 2008 [18]	Int J Med Sci	1) 851	Lung cancer chemoprevention trial	Yes/No	1) 23.5 ​% yes [20.7, 26.3]
		2) 396			2) 26.8 ​% yes [22.4, 31.2]
Mancini J, 2007 [31]	Contemp Clin Trials	188	New vs old chemotherapy regimen for breast cancer for cancer patients	5-point Likert scale (Yes absolutely, Yes maybe, …, No not at all)	Yes, absolutely: 23.8 ​% [17.7, 29.9]
					Yes, maybe: 19.2 ​% [13.6, 24.8]
Martin-McGill KJ, 2017 [32]	Seizure	102	Ketogenic diet vs no diet for refractory epilepsy	Willing/Unwilling	36.3 ​% willing [27.0, 45.6]
Mayanja Y, 2020 [21]	BMC Public Health	92	HIV Vaccine vs placebo	Yes/No	90.2 ​% yes [84.1, 96.3]
Palmer AJ, 2013 [13]	Bone Joint Res	31	Non-operative vs operative treatment of femoroacetabular impingement	Willing/Unwilling	90 ​% willing [79.4, 100]
Piazza A, 2019 [14]	Contraception	138	Salpingectomy vs tubal occlusion for contraception	Yes/No	39.1 ​% yes [31.0, 47.2]
Shah A, 2012 [33]	Int J Radiat Oncol Biol Phys	46	Radiotherapy vs proton therapy for proton cancer	Likely/Unlikely	58.7 ​% likely [44.5, 72.9]
Solomon MJ, 2003 [9]	Surgery	100	1) Open vs. Laparoscopic resection	Willing/Unwilling	1) 45 ​% willing [35.2, 54.8]
			2) Colostomy vs. Local resection		2) 45 ​% willing [35.2, 54.8]
			3)Surgery with chemotherapy vs. surgery alone		3) 45 ​% willing [35.2, 54.8]
			4) surgery with colostomy vs chemoradiotherapy without surgery		4) 31 ​% willing [21.9, 40.1]
Spierenburg W, 2022 [15]	Injury	100	1) surgery vs cast of distal radius fracture	5-point Likert scale (definitely want, probably do, …, definitely do not want)	1) 66 ​% definitely or probably willing [56.7.4, 75.3]
			2) CT-Scan vs no CT-scan for distal radius fracture		2) 68 ​% definitely or probably willing [58.9, 77.1]
Summers RH, 2015 [16]	Obes Surg	614	Non-operative vs bariatric surgery for Type-2 diabetes mellitus	3-item scale (yes, maybe, no)	30 ​% yes [26.4, 33.6]
Tsoi D, 2011 [34]	Breast	348	Non-surveillance vs surveillance of breast cancer	5-point Likert scale (strongly agree, agree, …, disagree)	31 ​% strongly agree [26.1, 35.9]
					27 ​% agree [22.3, 31.7]
Turner CE, 2008 [17]	Aust N Z J Obstet Gynaecol	102	Vaginal delivery vs. C-sections	3-item scale (yes, maybe, no)	14 ​% yes [7.3, 20.7]
Waldman AT, 2011 [22]	Front Neurol	198	Intravenous corticosteroids vs oral corticosteroids vs oral placebo for optic neuritis and multiple sclerosis	Yes/No	21 ​% yes [15.3, 26.7]
Walker KF, 2023 [10]	Health Technol Assess	259	Fetal pillow vs vaginal push technique for impacted fetal head	5-point Likert scale (most likely, …, least likely)	37.8 ​% most likely or likely [31.9, 43.7]
Weinfurt KP, 2017 [48]	Med Care	839	No Specified Intervention	Yes/No	N/A^a^

a Willingness reported as a percentage range based on numerous vignette designs.

b No specified intervention means the study asked generically about participating in an RCT, not in relation to a specific intervention.

c Willingness reported as an average score between 0 and 10, no proportion of participants' willingness assessed.

Three papers (8 ​%) did not report an overall WTP percentage and instead reported WTP either as an average value scored between one to ten or a WTP percentage range between multiple vignette designs (no individual percentage was reported). Four papers (10 ​%) reported more than one WTP percentage either due to multiple subgroups (e.g. male and female) or multiple proposed interventions (e.g. surgical and diagnostic RCT, vaccine and drug RCT), leading to an overall 44 WTP measurements from 37 PPAs. Participants’ WTP in each hypothetical trial from the 37 PPAs ranged from 13 ​% to 92.4 ​% with a median WTP of 54.9 ​%. When restricted to “definitely willing” responses, median willingness among the PPAs was 42.1 ​% (range: 7 ​%–90.2 ​%).

Among intervention types, eight papers [9,[11], [12], [13], [14], [15], [16], [17], [18]] proposed an operative vs non-operative intervention and had a median total willingness of 42.6 ​% (range: 14 ​%–90 ​%) (Table 2). Six papers [5,[18], [19], [20], [21], [22]] proposed drug vs placebo intervention and had a median total willingness of 27.8 ​% (range: 21 ​%–89.4 ​%) Seventeen papers [6,7,9,10,15,[23], [24], [25], [26], [27], [28], [29], [30], [31], [32], [33], [34]] proposed a design other than operative vs. non-operative or drug vs. placebo and had a median total willingness of 50.7 ​% (range: 13 ​%–92.4 ​%). When restricting to “definitely willing” responses, PPAs with an operative vs. non-operative intervention had a median willingness of 30.5 ​% (range: 14 ​%–90 ​%); PPAs with a drug vs. placebo intervention had a median willingness of 27.8 ​% (9 ​%–90.2 ​%); and PPAs with interventions other than operative vs. non-operative or drug vs. placebo had a median willingness of 41 ​% (7 ​%–68 ​%).

**Table 2:** Median willingness by intervention type.

Intervention Type	Number of studies (n)	Median (range) Definitely Willingness (%)	Median (range) Total Willingness (%)
Non-operative vs operative	8	30.5 (14–90)	42.6 (14–90)
Drug vs. Placebo	6^a^	27.8 (9–90.2)	27.8 (21–89.4)
Other	17^b^	41.4 (7–68)	50.7 (13–92.4)

a 8 reported WTP values.

b 20 reported WTP values.

In 12 PPA papers, the authors commented on whether a trial seemed feasible based on the PPA results. In six cases [10,11,13,16,32,34], authors stated that a trial would be feasible due to reasons such as high participant willingness and positive qualitative responses. Two papers [28,35] stated that changes were needed to conduct a trial. Four papers [17,19,24,26] concluded that a trial was not feasible; reasons included treatment preference by PPA participants or low participant willingness.

### 3.3 Association between PPAs and trials

Of the 29 eligible PPAs with a specified intervention and WTP data, we linked ten (34.5 ​%) to a registered RCT (Table 3). One study [11] was followed up with a clinical trial registered in the Australian New Zealand Clinical Trials Registry (ACTRN12615001177549) and the other nine were registered in ClinicalTrials.gov. Of the ten PPA-connected trials, nine trials have additional information published beyond their clinical trial registration including five trial manuscripts with results [[36], [37], [38], [39], [40]], two abstracts from annual meetings [41,42], and two trial protocol manuscripts [43,44]. While one of the two trials with a protocol paper is not yet published [44], we had access to the enrollment data. Thus, six RCTs (five published, one unpublished) had enrollment results [[36], [37], [38], [39], [40]] available to compare to the corresponding PPA. Among the six PPAs with RCT enrollment data, median total willingness was 52.4 ​% (range: 13 ​%–90 ​%). Among the 23 PPAs without RCT enrollment data (and that specified an intervention and reported percent WTP) median total willingness was 54.7 ​% (range: 14 ​%–92.4 ​%).

**Table 3:** PPA studies and connected registered clinical trials.

PPA	Clinical Trial Number	Trial Publication
Boye GN, 2015 [11]	ACTRN12615001177549^a^	**Hunter DJ, 2021** [36]
Creel AH, 2005 [12]	NCT00597012	**Katz JN, 2013** [37]
Dolan L, 2008 [26]	NCT00448448	**Weinstein SL, 2013** [39]
Hyung B, 2022 [6]	NCT05722002	N/A
Israni AK, 2004 [28]	NCT00264758	**FHN Trial Group, 2010** [38]
Kasiske BL, 2011 [7]	NCT03674307	Ying T, 2019 [43]
Kerman HM, 2017 [29]	NCT03059004^b^	**Sullivan JK, 2018** [44]
Palmer AJ, 2013 [13]	NCT01893034	**Palmer AJR, 2019** [40]
Shah A, 2012 [33]	NCT01617161	Wisdom A, 2023 [41]
Tsoi D, 2011 [34]	NCT03881605	Jerzak KJ, 2021 [42]

∗Trials with results are bolded.

a RCT registered in the Australian New Zealand Clinical Trials Registry.

b No publication found from registered RCT – recruitment is on-going.

**Fig. 2:**
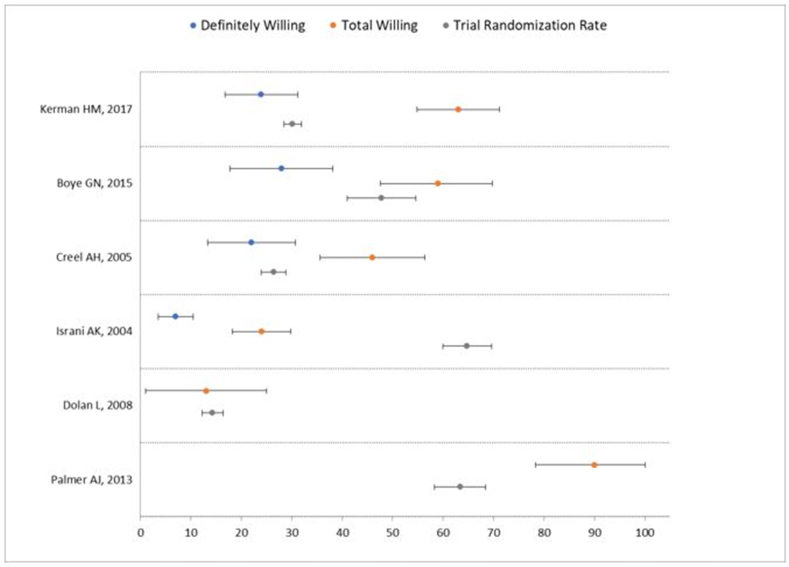
Graphical representation of willingness data of the six PPAs connected to a trial with enrollment data. Data points indicate definitely willing, total willing, and trial enrollment percentages with 95 ​% confidence intervals as their upper/lower bounds. PPAs utilizing Likert scales aggregated “definitely willing” and “probably willing” percentages into a “Total Willing” percentage.

To gain insight into the predictive accuracy of the hypothetical PPA, we compared WTP documented in six PPAs with actual randomization rates from the trial enrollment data (Table 4). Four of six PPAs [11,12,28,29] estimated willingness through the response options: “definitely willing”, “probably willing”, “unsure”, “probably unwilling”, “definitely unwilling”. In three of these four PPAs, the trial randomization rate lay between the definite and total estimates (Fig. 2). Two of the six PPAs estimated willingness using a binary scale (willing vs. unwilling). In one of these trials, the total willing in the PPA (13 ​%) was nearly the same as the percent of eligibles randomized in the actual RCT (14 ​%). The other PPA using a binary willing vs unwilling outcome overestimated the percent of eligibles who enrolled (90 ​% vs 63 ​%).

**Table 4:** PPA willingness data in Comparison and connected RCT enrollment data.

PPA Paper	Intervention Proposed	Sample Size	Definitely Willing [95 ​% CI]	Probably Willing [95 ​% CI]	Total Willing^c^ [95 ​% CI]	Trial Publication	Eligible Subjects (N)	Randomization Rate^b^ [95 ​% CI]
Palmer AJ, 2013 [13]	Non-operative vs operative treatment of femoroacetabular impingement	31	N/A^a^	N/A^a^	90 ​% [79.4, 100]	Palmer AJR, 2019 [40]	350	63.4 ​% [58.4, 68.5]
Kerman HM, 2017 [29]	Home exercise vs in-person physical therapy vs topical treatment for meniscus tear treatment	134	24 ​% [16.8, 31.2]	39 ​% [30.7, 47.3]	63 ​% [54.8, 71.2]	Unpublished^d^	2891	30.2 ​% [28.6, 31.9]
Boye GN, 2015 [11]	Non-operative vs operative treatment of femoroacetabular impingement	75	28 ​% [17.8, 38.1]	31 ​% [20.3, 41.1]	59 ​% [47.6, 69.8]	Hunter DJ, 2021[36]	207	47.8 ​% [41.0, 54.6]
Creel AH, 2005 [12]	Non-operative vs operative treatment of meniscal tears	88	22 ​% [13.3, 30.7]	24 ​% [15.1, 32.9]	46 ​% [35.6, 56.4]	Katz JN, 2013[37]	1330	26.4 ​% [24.0, 28.8]
Israni AK, 2004 [28]	Daily vs conventional hemodialysis	209	7 ​% [3.5, 10.5]	17 ​% [11.9, 22.1]	24 ​% [18.2, 29.8]	FHN Trial Group, 2010 [38]	378	64.8 ​% [60.0, 69.6]
Dolan L, 2008 [26]	Bracing vs observation for adolescent idiopathic scoliosis	24	N/A^a^	N/A^a^	13 ​% [0, 26.5]	Weinstein SL, 2013 [39]	1086	14.3 ​% [12.2, 16.4]

a Positive willingness response options (definitely willing, probably willing, etc.) were not stratified. Percentage reported in Total Willingness.

b Rate calculated from the proportion of participants randomized to participants eligible.

c Total Willingness derived from sum of “Definitely Willing” and “Probably Willing” percentages.

d Unpublished enrollment data from TeMPO trial (NCT03059004) provided by Katz JN, 2024.

## 4 Discussion

We sought to identify published PPAs, describe their salient features, and determine their predictive accuracy by comparing WTP data from the PPA with randomization rates of the actual corresponding RCTs. We identified 40 eligible papers that provided WTP data. There were no appreciable differences in WTP in the hypothetical trial among types of interventions (operative vs. non-operative, drugs vs. placebo, etc.). We found that ten of these 40 published PPAs were associated with a registered clinical trial, and six PPAs were followed by RCTs that provided trial enrollment data. The actual enrollment lay between “definitely willing” and “total willing” in three of these four PPAs. We are not aware of prior assessments of the predictive accuracy of PPAs willingness data for RCT enrollment.

The PPAs published to date vary in how they elicit WTP. It is unclear which approach is ideal. We observed that four PPAs with corresponding completed RCTs offered graded responses in a Likert format including “definitely willing” and “probably willing.” Our analysis suggests that the “definitely willing” percentage and combining “definitely willing” and “probably willing” percentages into a “total willing” percentage may provide lower and upper bounds of estimated trial enrollment. However, with a sample size of only four trials, this finding should be interpreted cautiously. Half of the registered RCTs identified (Table 2) were in their early stages with limited/no enrollment data when we gathered data for this study. These trials will eventually publish enrollment data, which can be compared to the corresponding PPAs to yield more robust estimates of predictive validity.

A limitation of our review is that only six PPAs were associated with RCTs that provided trial enrollment data. Thus, our ability to estimate the predictive validity of the PPA should be regarded as hypothesis generating. Also, our search yielded ten studies that assessed WTP in generic RCTs with no specification for an intervention. These studies were intended to assess the effect on WTP of features of a clinical trial, including populations [45,46] or consent procedures [47,48].

Another limitation of our review involves variability in WTP percentages ranging from 9 ​% to 92 ​%. This wide range may be explained by the heterogeneity of the proposed interventions, sample size, and study populations in the 40 PPAs. The effect of these factors is best seen in Palmer et al., where a larger sample size and more stringent eligibility criteria in the clinical trial yielded an overestimation of the PPA's WTP compared to the trial randomization rate [13,40]. While our data suggest it would be reasonable to use the upper and lower bounds of willingness in the PPA to estimate willingness in the actual trial, our findings also show that this guidance could potentially lead investigators astray [7,28]. As the literature on PPAs grows, the community will be able to determine whether departures from the upper and lower bounds occur rarely or more frequently.

In addition to written questionnaires assessing subjects' WTP in a hypothetical trial, Halpern et al. suggests incorporating a qualitative component to elicit participants' understanding of the trial design, open-ended questions to evaluate a participant's motivations, and written questionnaires to assess participants' willingness to participate [3]. Three of the seven PPAs with a qualitative analysis cited the qualitative data contributed to the determination of trial feasibility. These findings suggest a mixed-methods approach in a PPA may provide complementary insights.

We conclude that the PPA is a promising method for estimating RCT feasibility and forecasting actual trial enrollment among its potential participant population. It is important to note that other factors, such as the trial funding source, participant's gender, and participant's cultural background may also influence enrollment beyond their willingness [49,50]. However, the findings we present in this paper offer preliminary evidence of the PPA's predictive validity, and we encourage further research linking PPAs to subsequent trials to assess validity with greater precision.

## Contributions

JD and JNK contributed to conception and design. All authors contributed to analysis and interpretation of data. JD and PO contributed to collection and assembly of data. JD and JNK contributed to drafting of article. All authors contributed to final approval of article.

## Funding source

Supported by: NIH/NIAMS grant 5R34AR082021, 1R01AR055557, P30AR072577, K01AR075879 (Dr. Collins).

## Competing interest statement

JC declares receiving support from Boston Imaging Core Labs, LLC and American College of Rheumatology. There are no other competing interests to declare.
